# FoSSA Optimization-Based SVM Classifier for the Recognition of Partial Discharge Patterns in HV Cables

**DOI:** 10.1155/2022/7566731

**Published:** 2022-03-25

**Authors:** Kang Sun, Yuxuan Meng, Shuchun Dong

**Affiliations:** ^1^School of Electrical Engineering and Automation, Henan Key Laboratory of Intelligent Detection and Control of Coal Mine Equipment, Henan Polytechnic University, Jiaozuo 454003, China; ^2^Dianrong Intelligent Technology Co., Ltd., Kunshan 215334, China

## Abstract

In order to enhance the classification accuracy and the generalization performance of the SVM classifier in cable partial discharge (PD) pattern recognition, a firefly optimized sparrow search algorithm (FoSSA) is proposed to optimize its kernel function parameters and penalty factors. First, the Circle-Gauss hybrid mapping model is employed in the population initialization stage of the sparrow search algorithm (SSA) to eliminate the uneven population distribution of random mapping. Sparrows tend to fall into local extremums during the search process, while the firefly algorithm has a fast optimization speed and strong local search ability. Thus, a firefly disturbance is added in the sparrow search process, and the fitness value is recalculated to update the sparrow position to enhance the sparrow's local optimization ability and accuracy. Finally, based on the SSA, a dynamic step-size strategy is adopted to make the step size dynamically decrease with the number of iterations and improve the accuracy of convergence. Six benchmark functions are employed to evaluate the optimization performance of the FoSSA quantitatively. Experiment results show that the recognition accuracy of the PD patterns using the SVM optimized by the FoSSA could reach 97.5%.

## 1. Introduction

Power cable is the key infrastructure equipment for urban distribution networks and large-scale clean energy access, and its reliability is vital to the safe and stable operation of the power system [1]. The defects of insulation material, manufacturing process, and structural defects, coupled with the aging of insulation material caused by harsh electrical, thermal, and mechanical stresses environment, will result in a partial discharge (PD) and even dielectric breakdown, which lead to insulation failure [2]. Accurate and instant identification of the fault pattern by mining and analyzing operation fault records and all types of test data of the power cable can significantly improve the efficiency for the maintenance and overhaul of a cable system.

Due to the low frequency of faults during operation, imperfect records of fault, and abnormal information, the size of fault samples is usually limited. Benefitting from the structural risk minimization (SRM) criteria and the kernel methods, support vector machines (SVMs) [3] have shown significant superiority to deal with the classification problems of few samples and nonlinear high-dimensional data. Thus, it has been widely applied in the fault pattern recognition of large-scale electrical equipment, such as cables, transformers, and power grids [4–7]. However, the classification performance of the SVM is highly dependent on the selection of kernel function parameters and penalty factors, so how to optimize the parameters is crucial for its further applications.

For deterministic optimization algorithms, such as sequential minimal optimization (SMO) [8] and stochastic gradient descent (SGD) [9], if the objective function is discontinuous and nondifferentiable, their convergence speed is usually slow and they will easily fall into the local optimum. As a stochastic optimization method, the swarm intelligence optimization methods introduce a brand new path to solve global optimization problems by taking advantage of randomness. The particle swarm optimization (PSO) algorithm [10] and the ant colony optimization (ACO) algorithm [11] are the most representative of these.

The PSO algorithm has few parameters and a fast convergence speed, but it tends to fall into local extremes due to premature convergence. This can be improved partly by introducing inertial weighting factors and taboo detection mechanisms. However, for complex high-dimensional problems, usually, it is impossible to guarantee convergence to the global optimum. The ACO algorithm uses the positive feedback mechanism of ant colony pheromones to strengthen the learning ability. Its heuristic probabilistic search mode makes it not easy to trap in the local optimum. However, the parameter settings are complicated and searching speed is slow; furthermore, the convergence property is pretty poor. In order to further strike a balance between the search range and the convergence accuracy in optimization algorithms, a series of bionic intelligent optimization algorithms, such as gray wolf optimization algorithm [12] (GWO), artificial bee colony algorithm [13] (ABC), and the bacterial foraging algorithm [14] (BFA), have been proposed in recent years. Sparrow search algorithm (SSA) [15] is a novel swarm intelligence optimization algorithm that is inspired by foraging and antipredation behaviors of sparrows. Testing results on unimodal and multimodal functions demonstrate its superiority over PSO, ACO, and GWO in terms of accuracy, convergence speed, stability, and robustness.

This paper proposes a FoSSA-optimized SVM for the recognition of partial discharge patterns in HV cables. First, the feature vector is constructed based on the partial discharge *φ* − *q* − *n* pattern. Second, in the standard SSA, the Circle-Gauss hybrid mapping model is introduced to initialize the population to improve the diversity. In the sparrow search process, the dynamic step strategy and the firefly interference strategy are introduced to make the sparrow escape from the local optimum and find the global optimal combination of support vector machine parameters. Finally, an optimized SVM classification framework is constructed for the partial discharge recognition in the HV power cables.

## 2. Problem Description

The basic principle of a nonlinear SVM is to map the input space *x* to a feature space Φ(*x*) through a nonlinear transformation which results in a hyperplane model in the feature space corresponding to the hypersurface model in the input space. The hyperplane in the feature space is divided as follows:

(1) $$\begin{array}{c} f\left(x\right)=\omega^{Τ}\Phi \left(x\right)+b, \end{array}$$

where *ω* is the weighting vector and *b* is the threshold.

For a given nonlinear separable data set, considering the existence of errors *ξ*, the optimization problem with the constraint conditions is as follows:

(2) $$\begin{array}{c} \min\frac{1}{2}{\left\|\overset{⟶}{\omega }\right\|}^{Τ}\omega +c\sum_{i=1}^{l}\xi_{i},\\ \text{st.}y_{i}\left({\overset{⟶}{\omega }}^{Τ}\Phi \left(x_{i}\right)-b\right)\geq 1-\xi_{i}\left(\xi_{i}>0\right). \end{array}$$

The optimization of equation (2) can be transformed into a dual problem by introducing the Lagrange factor, and the solution of equation (1) can be obtained as follows:

(3) $$\begin{array}{c} f\left(x\right)=\sum_{i=1}^{l}\alpha_{i}\kappa \left(x,x_{i}\right)+b, \end{array}$$

where *α*_*i*_ is the Lagrange factor, *l* is the number of support vectors, and *κ*(*x*, *x*_*i*_) is the kernel function. A radial basis function (RBF) in equation (4) is generally adopted.

(4) $$\begin{array}{c} \kappa \left(x,x_{i}\right)=\exp\left(-g{\left\|x-x_{i}\right\|}^{2}\right). \end{array}$$

It can be seen from the derivation that the parameter selection of *c* and *g* directly affects the classification performance of SVM. In the traditional SVM model, they are usually selected according to expert experience or k-fold cross verification. In the process of cable fault classification, the input data are diverse and complex. The parameter selection based on experience not only takes time but also brings some randomness to the calculation process. The *K*-fold cross-verification method is dependent on the parameter range; if this range is inappropriate, it is impossible to determine the optimal parameters.

## 3. Firefly Optimized Sparrow Search Algorithm (FoSSA)

### 3.1. SSA Principle

SSA is a novel swarm intelligence algorithm that has evolved from the foraging and antipredation behaviors of sparrows. The algorithm is simple and efficient, and it can achieve global convergence. According to the mathematical model of the algorithm, virtual sparrows are used for foraging behavior and the position of sparrows can be expressed as follows:

(5) $$\begin{array}{c} X=\left[\begin{array}{c} X_{1}\\ X_{2}\\ \vdots \\ X_{n} \end{array}\right]=\left[\begin{array}{cccc} x_{1,1} & x_{1,2} & \cdots & x_{1,d}\\ x_{2,1} & x_{2,2} & \cdots & x_{2,d}\\ \vdots & \vdots & \vdots & \vdots \\ x_{n,1} & x_{n,2} & \cdots & x_{n,d} \end{array}\right], \end{array}$$

where *d* is the dimension of the variable in the optimization question, *n* is the number of sparrows, and *x*_*i*,*j*_ is the position of the *i*-th sparrow of the *j*-th dimension.

The fitness value of all sparrows can be calculated as the following vector:

(6) $$\begin{array}{c} F_{x}=\left[\begin{array}{c} f\left(x_{1,1}\text{, }x_{1,2}\text{, }...\text{, }x_{1,d}\right)\\ f\left(x_{2,1}\text{, }x_{2,2}\text{,}\ldots \text{, }x_{2,d}\right)\\ \vdots \\ f\left(x_{n,1}\text{, }x_{n,2}\text{,}\ldots \text{, }x_{n,d}\right) \end{array}\right], \end{array}$$

where *f* represents the individual fitness value.

A sparrow population can be divided into producer and scrounger according to the relative role of each sparrow. Producers are in charge of looking for food for the whole population; they provide foraging directions for the scroungers. Producers can obtain a larger foraging area than the scroungers. According to the foraging rules of sparrow population, the moving position of the producer is calculated as follows:

(7) $$\begin{array}{c} X_{i}^{t+1}=\left\{\begin{array}{cc} X_{i}^{t}\cdot e^{-\left(i/\alpha .t_{\max}\right)} & \text{if }V_{a}<V_{s},\\ X_{i}^{t}+r_{d}\cdot U & \text{if }V_{a}\geq V_{s}, \end{array}\right. \end{array}$$

where **X**_*i*_^*t*^ is the position of the *i*-th sparrow in *j*-dimension space at iteration *t*, *i* ∈ [1,2,…, *n*], *t* indicates the current iteration, *t*_max_ is the maximum iteration number, *α* ∈ (0,1] is a random number, *r*_*d*_ is a random number that obeys a normal distribution, *U* represents a unit matrix of 1 × *d*, and *V*_*a*_ ∈ [0,1] and *V*_*s*_ ∈ [0.5, 1] are the alarm and safety value relative to the predators, respectively, and they determine the sparrow's moving range.

During the foraging process, the scroungers keep eyes on the producers. Once the producers find something better, they fight for it immediately. According to the rules, their moving position can be updated as follows:

(8) $$\begin{array}{c} X_{i}^{t}=\left\{\begin{array}{cc} r_{d}\cdot e^{\left(X_{wst}^{t}-X_{i}^{t}\right)/i^{2}} & \text{if }i>n/2,\\ X_{op}^{t+1}+\left\|X_{i}-X_{op}^{t+1}\right\|\cdot A^{+}\cdot U & \text{others,} \end{array}\right. \end{array}$$

where **X**_*op*_ is the optimal location of the producers, **X**_*wst*_^*t*^ is the current global worst location, **A** is the matrix of 1 × *d* with elements are all 1 or −1 randomly, and **A**^+^=**A**^Τ^(**AA**^Τ^)^−1^.

In the search process, some sparrows called guards will be aware of the danger from the predators, according to the antipredation rule, and the mathematical expression of their moving positions can be obtained as follows:

(9) $$\begin{array}{c} X_{i}^{t+1}=\left\{\begin{array}{cc} X_{bst}^{t}+\beta .\left\|X_{i}^{t}-X_{bst}^{t}\right\| & \text{if }f_{i}>f_{b},\\ X_{i}^{t}+\gamma .\left(\frac{\left\|X_{i}^{t}-X_{wst}^{t}\right\|}{\left(\left(f_{i}-f_{w}\right)+\varepsilon \right)}\right) & \text{if }f_{i}=f_{b}, \end{array}\right. \end{array}$$

where **X**_*bst*_^*t*^ is the current global optimal value, *β* is a random parameter obeying standard normal distribution that constraints step size, *γ* ∈ [−1,1] is a random number, *f*_*i*_ is the fitness value of the current sparrow, *f*_*b*_ and *f*_*w*_ are the best and worst fitness value, and *ε* is a regulatory factor.

### 3.2. Initialization by Circle-Gauss Hybrid Mapping

The distribution of the initial population is important for SSA. A uniform and fully mapped initial distribution will effectively improve the convergence speed of the optimization process. Due to the lack of initialization strategy for uniform population distribution in SSA, simple random distribution cannot guarantee the breadth of the search range, and it is easy to produce “super sparrows” in the iterative process that cause other individuals to gather to them, resulting in a “premature” phenomenon and reducing the diversity of the population.

In this study, a Circle-Gauss hybrid mapping model is introduced to initialize the SSA. By combining the advantage of the regularity and uniformity of Circle mapping and the randomness and ergodicity of Gauss mapping, the chaotic sequence can be transformed into the solution space of the SSA algorithm to replace the original population by Circle-Gauss hybrid mapping model. The Circle-Gauss hybrid mapping model not only avoids the overdensity of the population but also retains the diversity of the population to a large extent, which is the key factor for the global optimization of the SSA algorithm. The mathematical expression of the Circle-Gauss hybrid mapping model is as follows:

(10) $$\begin{array}{c} M_{i+1}=\left\{\begin{array}{cc} M_{i}+a-\mathrm{mod}\left(\frac{b}{2\pi }\sin\left(2\pi M_{i}\right),1\right) & \text{if }i=2k\text{;}\\ \frac{1}{M_{i}}-\left[\frac{1}{M_{i}}\right] & \text{if }i=2k+1\text{;} \end{array}\right., \end{array}$$

where **M**_*i*_ ∈ *ℝ*^1×*d*^ is the mapping position of *i*-th sparrow, **M**=[**M**_1_, **M**_2_,…,**M**_*n*_]^*T*^ ∈ *ℝ*^*n*×*d*^ is the mapping position of the whole population, *n* and *d* have the same meaning as in equation (5). *a* is 0.5, *b* is 2.2, and *k*=0,1,2.... mod() and represent modulation and rounding operation.

**Figure 1:**
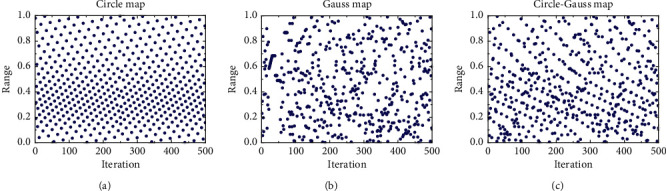
Population initialization scatter plot of three methods. (a) Circle map. (b) Gauss map. (c) Circle-Gauss map.

Figure 1 shows the 2D scatter diagram generated by the Circle-Gauss hybrid mapping model and the other two mappings in (0, 1). As shown in Figure 1, the Circle-Gauss hybrid mapping model combines the characteristics of both uniformity and randomness, improves the ergodicity and effectiveness of the initialization, and ensures the diversity of the population.

### 3.3. Firefly Perturbation

Firefly perturbation is introduced in SSA here to improve its global convergence ability while the sparrow falls into the local extremum. In the two-dimensional solution space of SVM parameter combination (*c*, *g*), analogous to the idea of the firefly algorithm, each sparrow is initialized by the attractiveness of *ρ*_0_. During the disturbance process, the degree of attraction decreases with the increase of the spatial distance. When the sparrow traps in a local optimum, the perturbation will be introduced as follows:

(1) The spatial distance *r* between the sparrow position **X**_*i*_^*t*^ and the optimal position **X**_*bst*_^*t*^ is calculated as follows:
  (11) $$\begin{array}{c} r=\frac{\sqrt{d_{m}}}{b_{l}-b_{u}}\left\|X_{i}^{t}-X_{bst}^{t}\right\|. \end{array}$$
In the expression, *b*_*l*_ and *b*_*u*_ represent the lower and upper boundary, respectively, and *d*_*m*_ is the space dimension.
(2) The attractiveness value *ρ* of each individual sparrow is calculated:
  (12) $$\begin{array}{c} \rho =\rho_{0}e^{-\theta r^{2}}. \end{array}$$
In the expression, *ρ*_0_ is the maximum attractiveness and *θ* is the attractiveness coefficient.
(3) The sparrow position with perturbations **X**_*i*_^*t*′^ can be obtained as follows:
  (13) $$\begin{array}{c} X_{i}^{t^{\prime }}=X_{i}^{t}+\rho \left(X_{i}^{t}-X_{bst}^{t}\right)+\tau \left(R_{d}-0.5\right). \end{array}$$

Here, *τ* is the step-size factor and **R**_*d*_ ∈ *ℝ*^1×*d*^ is a matrix with all elements obeying uniform distribution within [0,1].

### 3.4. Dynamic Step-Size Updating

In the SSA, the step-size control parameters are constant, which cannot make the SSA achieve a balance between the local optimization and the global optimization in the iterative process. It will affect the effect and speed of the optimization.

In the initialization process, a longer step-size factor is adopted to enhance the algorithm's global search capability. In the later process, a smaller step is adopted to enhance the local optimization capability. Therefore, in this study, a dynamic updating strategy of step size is adopted to make the step size decrease nonlinearly with the increase of iteration number for the guards in equation (9). The dynamic step size can be optimized as follows:

(14) $$\begin{array}{c} \lambda \left(t+1\right)=i_{m}\lambda \left(t\right)e^{t/i_{m}}\left(\frac{\lambda_{0}}{\ln\;t}+r_{d}\left(1-\lambda_{0}\right)\right), \end{array}$$

where *λ*_0_ is the initial step-size factor and *i*_*m*_ is the maximum iteration number.

## 4. Cable PD Pattern Recognition Based on FoSSA-SVM

### 4.1. Feature Vector Extraction

Phase resolved partial discharge (PRPD) spectrum reveals the relationship between the number of PD signals with different peak values and the phase angle. Since the PRPD spectra of different defects achieve different distribution characteristics, statistical characteristic parameters based on PRPD can be used to recognized different insulation faults. In this study, 14 statistical characteristic parameters are employed for the feature extraction of the PD data. The expressions and the meanings of the characteristic parameters are shown in Table 1.

According to the calculation of statistical characteristic parameters, 9 key features are extracted as PD patterns, as shown in Table 2.

The skewness *S*_*k*_ reflects the skewness of the spectrum shape compared with the normal distribution. The steepness *K*_*u*_ is used to describe the protrusion degree of the distribution of a shape compared with the normal distribution shape. The factor *Q* reflects the difference of the average discharge in the pos(+) and neg(-) half-cycle of the *φ* − *q* spectrum. The phase asymmetry degree ∅ reflects the difference of the initial phase of the discharge within different half-cycles of the *φ* − *q* spectrum. The cross-correlation coefficient *C*_*c*_ reflects the degree of shape similarity of the spectra in different half-cycles.

### 4.2. PD Pattern Recognition Based on the FoSSA-SVM Model

**Figure 2:**
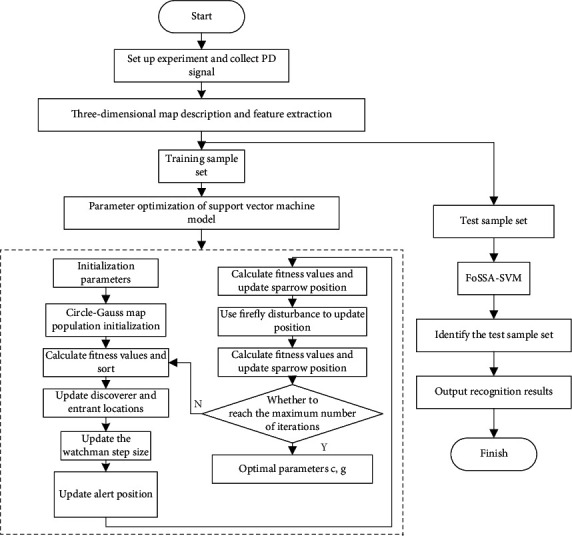
Flow chart of PD pattern recognition based on FoSSA-SVM.

The FoSSA-optimized SVM model for cable PD pattern recognition proposed in this paper is shown in Figure 2. The whole scheme can be divided into feature extraction, parameter optimization, and pattern recognition:

1. Four kinds of PD defects are manually prepared for data acquisition on a test platform. Then, the 3D PRPD spectrum is drawn for key feature extraction using statistical characteristics.
2. The parameters of FoSSA and SVM are initialized, and the penalty factor and kernel function parameters of SVM are taken as the optimization objective of FoSSA. During the initialization stage, the Circle-Gauss hybrid mapping model is employed to initialize the population of sparrows.
3. FoSSA algorithm is used for parameter optimization. The recognition error rate is set as the objective function for iterative calculation. The optimal parameter combination obtained is imported into the SVM model and verified by the test set. The detailed steps of the FoSSA to optimize the combination parameters are as follows:

  Step 1: the size of sparrow population, number of iterations, producer and scrounger ratios, step-size parameters, and SVM parameters are initialized.
  Step 2: the Circle-Gauss hybrid mapping model is used to generate the initial population of sparrows.
  Step 3: the fitness value is computed and sorted.
  Step 4: the positions of producers and scroungers are updated according to equation (7) and equation (8), respectively.
  Step 5: the step size of the guards is updated according to equation (14).
  Step 6: the position of the guards is updated according to equation (9).
  Step 7: update the sparrow positions taking consideration of firefly disturbance according to equation (13).
  Step 8: the fitness value is recalculated and the sparrow's position is updated.
  Step 9: if the stop conditions are met, the algorithm is exited and the results are output. Otherwise, repeat Steps 3–8.

## 5. Experimental Results and Analysis

### 5.1. PD Sample Set Construction

**Figure 3:**
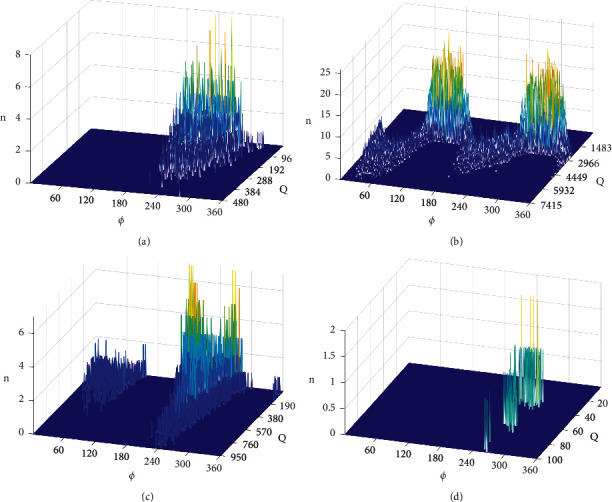
PRPD spectra of four type defects. (a) Outer semiconductive layer creepage (5.6 kV). (b) Internal air gap (5.3 kV). (c) Scratch of the insulation surface (5.6 kV). (d) Metallic filth (6 kV).

According to the designed experiment, the cable PD data are acquired. The sampling rate of the oscilloscope used in the experiment is set at 10 MS/s, and the sampling length is 1s each time; that is, the signal containing 50 power frequency cycles is taken as one sample. The number of samples from each type of defect is 50; that is, each PD defect contains 50 samples. The voltage levels corresponding to the measurement of the four PD defects are shown in Table 3. After the collection of sampling points, the PRPD spectra with four defects are drawn, as shown in Figure 3. The training set is 80% of these defect samples and the test set is 20%.

### 5.2. Comparison and Analysis of Convergence Performance

#### 5.2.1. Transverse Comparison and Analysis

Compared with other traditional algorithms such as PSO and GA, the FoSSA has obvious improvements in the convergence speed and optimization accuracy. In this study, three thirty-dimensional test functions are used to compare the convergence speed and optimization ability of FoSSA, GA, PSO, and GWO algorithms. The expressions of the test functions are shown in Table 4.

**Figure 4:**
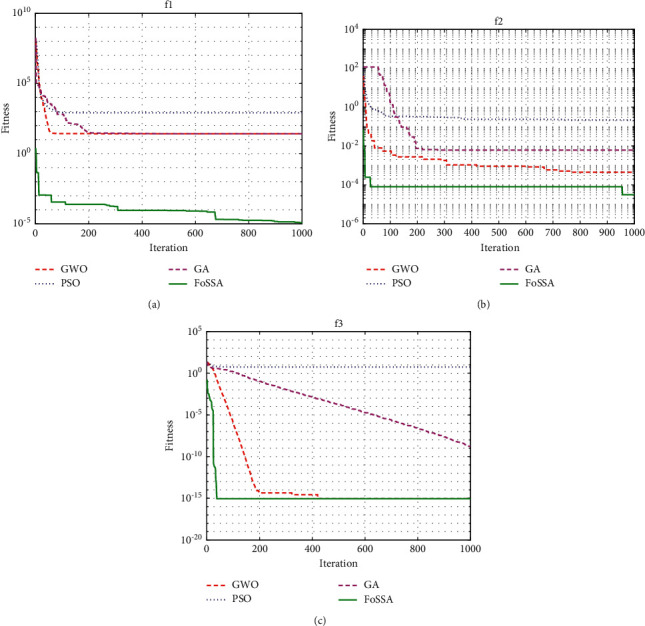
Convergence results of test functions. (a) Convergence results of *f*_1_(*x*). (b) Convergence results of *f*_2_(*x*). (c) Convergence results of *f*_3_(*x*).

To make the results more convincing, each test function is tested 30 times independently. The population is set to 100 and the maximum number of iterations is 1000. For FoSSA, the accounts of producers and sparrows aware of danger are set as 20% and 10% of the whole population and *v*_*s*_ is 0.8. Crossover probability *c* is 0.9, mutation probability *μ* is 0.03 in GA, *ω*=0.728 and *c*_1_=*c*_2_=1.49442 in PSO, *r*_1_, *r*_2_=random(0,1), and *a* ∈ (0,2) with a linear decrease in GWO. The optimization results of the four algorithms on the test function are shown in Figure 4.

The minimum values of the test functions are all zero. The results show that the PSO method has a fast convergence speed, but the convergence accuracy is very low. The GA and GWO are prone to local optimality. The FoSSA achieves the best convergence speed and convergence accuracy at the same time.

#### 5.2.2. Longitudinal Comparison and Analysis

SSA, LevySSA[16], RandSSA [17], and tSSA [18] are studied separately to compare with our FoSSA to demonstrate the optimization effect and convergence ability. For SSA, the accounts of producers and sparrows aware of danger are set as 20% and 10% of the whole population and the safety threshold value *V*_s_ is 0.8. For FoSSA, additional parameters of the step-size factor *τ* is 0.2 and the maximum attractiveness *ρ*_0_ is 2. The parameter *β* in LevySSA is 1.5. The parameter *r*(*t*) in RandSSA is set as 0 or 1 randomly. The parameter *p* in t-SSA is 0.5.

In this study, a six-dimensional single-peak function is employed to test the optimization ability of the function, and two thirty-dimensional multimodal functions are employed to test the ability to escape from the local optimum. The three test functions are shown in Table 5.

**Figure 5:**
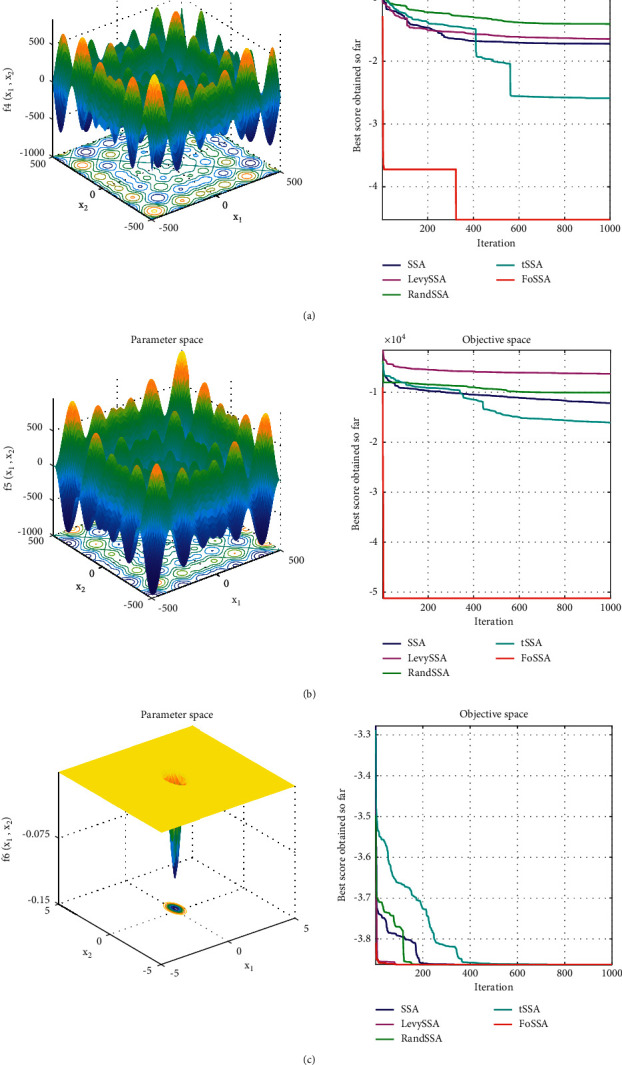
Convergence rate and optimal contour of test functions. (a) Convergence rate and optimal contour of *f*_4_(*x*). (b) Convergence rate and optimal contour of *f*_5_(*x*). (c) Convergence rate and optimal contour of *f*_6_(*x*).

Figure 5 shows the comparative analysis effect of five optimization algorithms on the minimum optimization of the test function. It is obvious that the FoSSA achieves the fastest convergence speed for the single-peak function of *f*_6_(*x*) and the strongest ability to transfer from local optimum for the multipeak functions of *f*_4_(*x*) and *f*_5_(*x*).

Each method optimizes 100 times for test functions, and the mean value, optimal value, and worst value are recorded, as shown in Table 6.

After comparing the FoSSA with the other four optimizers, it is found that the FoSSA obtains the strongest performance to get over the local optimum for the multipeak function and the fastest convergence speed of the single-peak function. The optimal value, worst value, and mean value of the FoSSA are the smallest, which means the best performance.

### 5.3. PD Pattern Recognition Results and Analysis

Based on the FoSSA algorithm, in order to find the optimal kernel function parameters *g* and the penalty factor *c*, the corresponding combination which achieves the minimum classification error rate after 30 iterations is chosen as the optimal parameter combination of *g* and *c*. The optimal parameter combination of different optimization algorithms is shown in Table 7.

**Figure 6:**
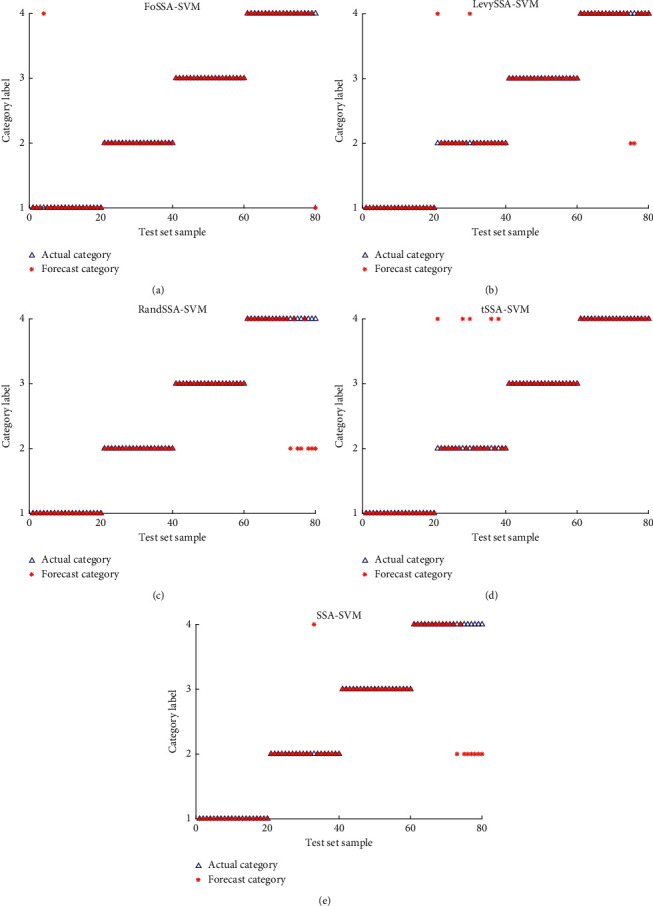
Classification results of different classification models. (a) FoSSA-SVM. (b) LevySSA-SVM. (c) RandSSA-SVM. (d) tSSA-SVM. (e) SSA-SVM.

The SSA-SVM, LevySSA-SVM, tSSA-SVM, RandSSA-SVM, and FoSSA-SVM classification models described in this paper are employed to recognize cable defects patterns with their optimal parameter combination. The results are shown in Figure 6 and Table 8.

Compared with the SSA-SVM, FoSSA-SVM improves the classification accuracy by 7.5%, and with the other classification models, it improves the accuracy by 2%–5%. In terms of time, the FoSSA-SVM requires the shortest iteration time, which is 32–150 ms shorter than that of other algorithms. In short, the FoSSA-SVM achieves a faster optimization speed and the highest recognition accuracy at the same time.

In order to further verify the prediction accuracy, the FoSSA-SVM model is compared with PSO-SVM and GA-SVM models. 80 samples are used for testing, and each model is employed to predict 30 times; the optimal prediction result is taken into account. The final results are shown in Table 9, in which it is obvious that the FoSSA-SVM model achieves a predictive accuracy of 97.5%, which is better than that of the other two algorithms.

The results of the experiment demonstrate that the proposed FoSSA-SVM model improves the prediction accuracy significantly in cable PD pattern recognition and it achieves obvious advantages in potential applications.

## 6. Conclusion

FoSSA is proposed in this paper to optimize the kernel function parameters and penalty factors of SVM for PD pattern recognition of cables. A novel Circle-Gauss hybrid mapping model used in the initialization stage of SSA improved the diversity of the sparrow population. Dynamic step-size and firefly disturbance strategy help SSA out of local optimum and then improve the convergence accuracy. Compared with SVM optimized by SSA, the classification accuracy is increased by 7.5% and the time consumption is shortened by 150 ms. The introduction of firefly perturbation and dynamic step strategy enhances the global convergence ability of SSA.

## Figures and Tables

**Table 1:** Expressions of characteristic parameters.

Symbol of parameters	Meaning
*sk*_*m*_^+^	*φ*−*q*_max_ pos(+) half-cycle skewness
*sk*_*m*_^−^	*φ*−*q*_max_ neg(−) half-cycle skewness
*sk*_*m*_	*φ*−*q*_max_ full-cycle skewnesss
*sk*_*n*_^+^	*φ*−*q*_mean_ pos(+) half-cycle skewness
*sk*_*n*_^−^	*φ*−*q*_mean_ neg(−) half-cycle skewness
*sk*_*n*_	*φ*−*q*_mean_ full-cycle skewness
*kt*_*m*_^+^	*φ*−*q*_max_ pos(+) half-cycle kurtosis
*kt*_*m*_^−^	*φ*−*q*_max_ neg(−) half-cycle kurtosis
*kt*_*m*_	*φ*−*q*_max_ full-cycle kurtosis
*kt*_*n*_^+^	*φ*−*q*_mean_ pos(+) half-cycle kurtosis
*kt*_*n*_^−^	*φ*−*q*_mean_ neg(−) half-cycle kurtosis
*kt*_*n*_	*φ*−*q*_mean_ full-cycle kurtosis
*r*_*m*_	*φ*−*q*_max_ ratio of the sum of pos(+) and neg(−) half-cycle discharge amplitudes
*r*_*n*_	*φ*−*q*_mean_ ratio of the sum of pos(+) and neg(−) half-cycle discharge amplitudes

**Table 2:** Symbol definition of characteristic quantity.

Characteristic	Symbol description
Mean	*μ*
Variance	*σ* ^2^
Skewness	*S*_*k*_
Steepness	*K*_*u*_
Local peak number	*P*_*e*_
Discharge factor	Q
Degree of phase asymmetry	∅
Cross-correlation coefficient	*C*_*c*_
Corrected cross-correlation coefficient	*m*_*c*_

**Table 3:** Discharge voltage of different defects.

Defect type	Discharge voltage
Outer semiconductive layer creepage	5.6 kV, 6.6 kV
Internal air gap	5.3 kV, 11.3 kV, 18.3 kV
Scratch of insulation surface	5.6 kV, 9.6 kV, 13.6 kV
Metallic filth on insulation surface	6 kV, 20 kV, 34 kV

**Table 4:** Test function expressions and their range.

Test function	Range
*f*_1_(*x*)=∑_*i*=1_^*n*−1^[100(*x*_*i*+1_ − *x*_*i*_^2^)^2^+(*x*_*i*_ − 1)^2^]	[−30, 30]
*f*_2_(*x*)=∑_*i*=1_^*n*^*ix*_*i*_^4^+random[0,1)	[−1.28, 1.28]
$f_{3}\left(x\right)=-20\;\exp\left(-0.2\sqrt{1/n\sum_{i=1}^{n}x_{i}^{2}}\right)-\exp\left(1/n\sum_{i=1}^{n}\cos\left(2\pi x_{i}\right)\right)+20+e$	[−32, 32]

**Table 5:** Function expressions and their range.

Test function	Range
$f_{4}\left(x\right)=\sum_{i=1}^{n}-x_{i}\sin\left(\sqrt{\left|x_{i}\right|}\right)$	[−500, 500]
$f_{5}\left(x\right)=\sum_{i=1}^{n}-x_{i}\cos\left(\sqrt{\left|x_{i}\right|}\right)$	[−500, 500]
*f*_6_(*x*)=−∑_*i*=1_^4^*c*_*i*_exp (−∑_*j*=1_^6^*a*_*ij*_(*x*_*j*_ − *p*_*ij*_))^2^	[0, 1]

**Table 6:** Comparison of optimization results.

Function	Optimizer	Optimal value	Worst value	Mean
*f*_4_(*x*)	SSA	−10139.98	−6154.46	−8145.43
	FoSSA	−36168.05	−28325.41	−34049.27
	LevySSA	−30725.72	−26342.56	−28072.35
	tSSA	−29325.62	−23210.74	−28705.26
	RandSSA	−23257.82	−19072.23	−21072.43

*f*_5_(*x*)	SSA	−15642.13	−13425.62	−14568.18
	FoSSA	−35653.03	−31971.04	−34895.24
	LevySSA	−16725.12	−14236.42	−15742.84
	tSSA	−16732.45	−14584.72	−15643.72
	RandSSA	−17325.42	−14346.28	−16435.65

*f*_6_(*x*)	SSA	−3.2432	−3.0898	−3.1042
	FoSSA	−3.8947	−3.8628	−3.8725
	LevySSA	−3.7243	−3.2649	−3.4634
	tSSA	−3.7254	−3.6234	−3.6927
	RandSSA	−3.3274	−3.1324	−3.2736

**Table 7:** Optimal parameter combination of different classification models.

Classifier	*c*	*g*
SSA-SVM	0.12	14.50
FoSSA-SVM	4.45	0.76
LevySSA-SVM	6.31	14.56
tSSA-SVM	1.77	8.11
RandSSA-SVM	0.14	8.34

**Table 8:** Comparison of accuracy and times consumption of different models.

Classifier	Accuracy	*t* (ms)
SSA-SVM	90%	512
FoSSA-SVM	97.5%	362
LevySSA-SVM	95%	494
tSSA-SVM	93.75%	394
RandSSA-SVM	92.5%	402

**Table 9:** Prediction accuracy of different models.

Classifier	FoSSA-SVM	PSO-SVM	GA-SVM
Error number	2	7	9
Accuracy	97.5%	91.25%	88.75%

## Data Availability

The data used to support the findings of this study are available from the corresponding author upon request.
